# New approaches to the effects of Si on sugarcane ratoon under irrigation in Quartzipsamments, Eutrophic Red Oxisol, and Dystrophic Red Oxisol

**DOI:** 10.1186/s12870-023-04077-2

**Published:** 2023-01-24

**Authors:** Milton G. Costa, Renato de M. Prado, Marcilene M. Santos Sarah, Luiz F. Palaretti, Marisa de C. Piccolo, Jonas P. Souza Júnior

**Affiliations:** 1 Faculty of Agricultural and Veterinarian Sciences, Department of Agricultural Production Sciences, São Paulo State University (UNESP), Jaboticabal, Via de Acesso Prof. Paulo Donato Castellane, São Paulo, 14884900 Brazil; 2 Nuclear Energy Center in Agriculture, University of São Paulo (USP), Av. Centenário, 303, Piracicaba, São Paulo, 13400-970 Brazil

**Keywords:** Beneficial element, *Saccharum officinarum* L., Homeostatic balance of C:N:P, Tropical soils, Fertigation with Si

## Abstract

**Background:**

C:N:P homeostasis in plants guarantees optimal levels of these nutrients in plant metabolism. H However, one of the causes to the effects of deficit irrigation is the loss of C:N:P homeostasis in leaves and stems that causes reduction in the growth of sugarcane. Being able to measure the impact of water deficit on C:N:P homeostasis in plants from the stoichiometric ratios of the concentrations of these nutrients in leaves and stems. This loss causes a decrease in nutritional efficiency, but can be mitigated with the use of silicon. Silicon favors the homeostasis of these nutrients and crop productivity. The magnitude of this benefit depends on the absorption of Si by the plant and Si availability in the soil, which varies with the type of soil used. Thus, this study aims to evaluate whether the application of Si via fertigation is efficient in increasing the absorption of Si and whether it is capable of modifying the homeostatic balance of C:N:P of the plant, causing an increase in nutritional efficiency and consequently in the production of biomass in leaves and stems of sugarcane ratoon cultivated with deficient and adequate irrigations in different tropical soils.

**Results:**

Water deficit caused biological losses in concentrations and accumulation of C, N, and P, and reduced the nutrient use efficiency and biomass production of sugarcane plants cultivated in three tropical soils due to disturbances in the stoichiometric homeostasis of C:N:P. The application of Si increased the concentration and accumulation of Si, C, N, and P and their use efficiency and reduced the biological damage caused by water deficit due to the modification of homeostatic balance of C:N:P by ensuring sustainability of the production of sugarcane biomass in tropical soils. However, the intensity of attenuation of such deleterious effects stood out in plants cultivated in Eutrophic Red Oxisols. Si contributed biologically by improving the performance of sugarcane ratoon with an adequate irrigation due to the optimization of stoichiometric ratios of C:N:P; increased the accumulation and the use efficiency of C, N, and P, and promoted production gains in biomass of sugarcane in three tropical soils.

**Conclusion:**

Our study shows that fertigation with Si can mitigate the deleterious effects of deficient irrigation or potentiate the beneficial effects using an adequate irrigation system due to the induction of a new stoichiometric homeostasis of C:N:P, which in turn improves the nutritional efficiency of sugarcane cultivated in tropical soils.

## Background

The use of irrigation systems has expanded in recent years aiming the cultivation of sugarcane. The main objective is to increase sugarcane productive potential, as irrigation systems increase the nutritional efficiency of fertilization, favoring physiological and biochemical processes and consequently the growth and development of the crop [1]. Thus, the scientific area of irrigated agriculture has gained significant momentum since the beginning of the twenty-first century [2]. This includes studies on sugarcane [3] because of the lengthening of water deficit periods during the crop growth cycle resulting from the aftereffects of climate change [4].

In parallel, the availability of “fresh” water suitable for irrigated systems has suffered a considerable decrease in recent years. However, the water demand for irrigation is expected to increase by 11% by 2050, which results in a greater challenge for many regions that live under water restrictions [2]. In addition, the fact that many irrigated systems depend on electricity and their high costs in energy have led to increases in expenditure in recent years [5].

This scenario indicates an urgent need for new approaches that can enhance the viability of irrigated sugarcane systems by increasing the production of stalks in crop areas with adequate irrigation or areas with deficient irrigation due to water scarcity. In the countryside, marginal regions with water deficit problems may reach 35% of the soil's water retention capacity [2] and cause a significant decrease in crop productivity. These effects can be aggravated in soils with low water retention capacity, such as Quartzipsamments. In this sense, it is fundamental to understand the water deficit in different soils, mainly tropical soils, such as latosol and Quartzipsamments.

Thus, water deficit is the main abiotic stress factor limiting sugarcane production worldwide, as it forms a strong relationship between growth rate and optimal soil moisture regimes [6, 7]. This indicates that sugarcane crops are responsive to irrigation and at the same time tolerant to lack of water [8]. The biological damages of water deficit to sugarcane are known, especially in the physiological processes of plants [9–12]. However, other biological damages have recently been reported due to changes in the homeostatic balance of C:N:P [13, 14].

A known and sustainable alternative to attenuate stress in crops is the beneficial element Si. It may also affect plants without stress [15–17]. An advantage of this element is its relatively low cost compared to nutrients present in conventional fertilizers [18]. It also poses no risk to the environment [19]. Studies carried out on Si in sugarcane crops attest to its benefits to plants grown under water deficit; those studies reported mainly the effect of Si on the water losses reduction, that is, transpiration [11, 20]. However, there is recent evidence that Si may affect nutritional components that are vital to the growth and development of sugarcane crops [21].

The first studies evaluating the relationships of Si in plant nutrition specifically considering the stoichiometry of C:N:P in sugarcane were carried out by our research group (Genplant) at Universidade Estadual Paulista (São Paulo, Brazil). The starting point was studies evaluating the beneficial effects of Si on elemental stoichiometry at the initial growth phase of pre-sprouted seedlings in sugarcane cultivated up to 30 days [13] and up to 80 days [14] in water deficit and in plants grown without stress for 90 days [22] and 125 days [16]. However, these studies were carried out using soilless cultivation in sand and nutrient solution, or in only one type of soil (Quartzipsamments). Thus, it is clear that this line of investigation is just beginning, and that there is a need for further works that aim to consolidate yet another benefit of Si which is still little explored. Therefore, considering that the Si absorption process is governed by several factors, such as genetics (cultivars) and availability of Si in the soil [21], it is possible that the effects of this beneficial element on plant elemental stoichiometry may be affected by soil types.

Tropical soils, despite having a high total Si content due to desilication process, have a relatively low available Si content in them (0.5 mol L^−1^ acetic acid extractor), reaching 5.4 mg dm^−3^ in Red Yellow Oxisol, 6.7 mg dm^−3^ in Dark Red Oxisol, and 2.7 mg dm^−3^ in Quartzipsamments [23]. Thus, strong crop responses to Si application are expected, especially in soils with a Si content below the critical level, that is, 15 mg dm^−3^ of Si (0.5 mol L^−1^ acetic acid extractor) [24] or 6–8 mg kg^−1^ (extractor in CaCl_2_ 0.01 mol L^−1^) [23]. However, soil types differ in mineralogical composition, texture, Fe and Al oxide contents, and organic matter [25]. These elements govern the availability of Si in the soil, which justifies the importance of studies considering different soils. These studies could be carried out with sugarcane ratoon, that is, the sprouting of the plant after the first cut or growth cycle. Information about Si at this crop stage is even scarcer.

Considering the above and given the need for a better understanding of the harmful underlying effects of water deficit especially in sugarcane ratoon, the ability of Si to reverse this stress and even improve the plant's stress-free response may be a new approach to study the benefits of Si in this species at the ratoon stage. For this, it is pertinent to test the following hypotheses: (i) initially verify whether sugarcane ratoon, without Si application, is sensitive to irrigation deficit due to disturbances in the stoichiometric homeostasis of C:N:P, leading to losses in the use efficiency of these nutrients and consequently contributing to the productivity of ratoon cultivated in three tropical soils; if this is confirmed, (ii) whether the use of Si can modify this element’s stoichiometry and mitigate the damage caused by water deficit by improving the nutritional efficiency of these nutrients, affecting the productivity of ratoon cultivated under deficient irrigation, and also (iii) consider adequate irrigation in three tropical soils.

This research evaluates these hypotheses with the objective of assessing whether the application of Si via fertigation is efficient in increasing the absorption of Si and whether it is capable of modifying the homeostatic balance of C:N:P of the plant, causing an increase in nutritional efficiency and consequently in the production of biomass in leaves and stalks of sugarcane ratoon cultivated with inadequate and adequate irrigation in different tropical soils.

## Results

### C, N, P and Si concentrations

Fertigation with Si increased the concentration of Si in sugarcane leaves and stems in the absence (AWD) and in the presence of water deficit (PWD) in Quartzipsamments, Eutrophic Red Oxisols, and Dystrophic Red Oxisols (Table 1). Water deficit, in turn, reduced the efficiency of Si fertigation compared to the condition of absence of water deficit. However, Si absorbed was sufficient to increase the beneficial element concentration in leaves and stems of sugarcane plants cultivated in three tropical soils. In n plants that did not receive Si via fertigation, water deficit decreased the ability of plants to absorb Si from the soil, leading to a decrease in the concentration of Si in leaves of sugarcane plants cultivated in Eutrophic Red Oxisols and Dystrophic Red Oxisols, while stems where only affected in plants grown in Eutrophic Red Oxisols (Table 1).

**Table 1:** C, N, P, and Si concentrations in leaves and stems of sugarcane plants in two water deficit conditions (presence: 35%, and absence: 70% of water retention) combined with absence (0.0 mmol L^−1^) and presence of fertigated Si (1.8 mmol L^−1^) in three tropical soils (Quartzarenic Neosol; Eutroferric Red Latosol; and Dystrophic Red Latosol)

Water deficit	Si	Quartzipsamments		Eutrophic Red Oxisols		Dystrophic Red Oxisols	
		**leaf**	**Stem**	**leaf**	**stem**	**leaf**	**stem**
C (g kg^−1^)							
Presence	without	437.27 Aa	413.52 Aa	437.35 Aa	425.81 Aa	428.55 Aa	422.71 Aa
	with	422.63 Ab	408.22 Aa	433.72 Aa	413.88 Ab	426.70 Aa	416.62 Ab
Absence	without	430.78 Aa	416.71 Aa	417.14 Ba	413.31 Ba	432.04 Aa	416.20 Ba
	with	417.98 Ab	406.6 Ab	416.77 Ba	414.39 Aa	419.92 Bb	411.59 Bb
N (g kg^−1^)							
Presence	without	4.37 Aa	3.67 Aa	8.89 Aa	5.17 Aa	6.25 Ba	4.22 Aa
	with	3.53 Bb	3.15 Ab	7.76 Ab	4.47 Ab	6.01 Aa	3.84 Aa
Absence	without	4.50 Aa	2.87 Ba	4.80 Ba	3.06 Ba	6.93 Aa	3.10 Ba
	with	4.05 Ab	2.48 Bb	4.26 Bb	2.53 Bb	5.33 Bb	2.52 Bb
P (g kg^−1^)							
Presence	Without	0.55 Bb	0.58 Bb	0.23 Bb	0.41 Bb	0.68 Bb	0.99 Bb
	With	0.63 Aa	0.62 Ba	0.27 Ba	0.48 Ba	0.71 Ba	1.12 Ba
Absence	Without	0.61 Aa	0.68 Ab	0.33 Ab	0.48 Ab	0.75 Ab	1.15 Ab
	with	0.64 Aa	0.71 Aa	0.38 Aa	0.54 Aa	0.85 Aa	1.42 Aa
Si (g kg^−1^)							
Presence	Without	2.81 Ab	1.97 Ab	3.64 Bb	3.13 Bb	3.44 Bb	2.36 Ab
	With	10.00 Ba	4.19 Ba	9.18 Ba	4.77 Ba	8.06 Ba	5.64 Ba
Absence	Without	3.41 Ab	2.41 Ab	5.55 Ab	5.66 Ab	4.85 Ab	3.41 Ab
	With	17.91 Aa	7.53 Aa	19.42 Aa	13.63 Aa	12.67 Aa	8.39 Aa

Different uppercase letters indicate differences in water deficit and different lowercase letters indicate differences in Si fertigation by Tukey test (*p* < 0.05)

C concentration in leaves and stems decreased in plants grown in Eutrophic Red Oxisols and in stems of plants grown in Dystrophic Red Oxisols in PWD and in the absence of Si fertigation (Table 1). Also, there was a decrease in C concentrations in leaves under the two water conditions studied in Quartzipsamments fertigated with Si; in Dystrophic Red Oxisols, there was a decrease only in the absence of water deficit (AWD); and in Eutrophic Red Oxisols, the treatments did not affect C concentration in leaves (Table 1). In stems, fertigation with Si decreased the concentration of C under both water conditions studied here in sugarcane cultivated in Dystrophic Red Oxisols; in Quartzipsamments, there was a decrease in C concentration only in AWD; and in Eutrophic Red Oxisols, the C concentration in stems decreased in the presence of water deficit (PWD) (Table 1).

Water deficit increased the N concentration in stems of sugarcane plants cultivated in the three tropical soils studied here in the absence of Si application. There was a similar response in leaves of plants cultivated in Eutrophic Red Oxisols. However, there was also a decrease in N concentration in leaves of plants grown in Dystrophic Red Oxisols in the absence of Si application (Table 1). Fertigation with Si also decreased the concentration of N in leaves and stems of plants under AWD and PWD in sugarcane cultivated in the three tropical soils. However, there was a decrease only in AWD in Dystrophic Red Oxisols (Table 1).

Water deficit decreased P concentration in leaves and stems of plants without Si fertigation in all three tropical soils (Table 1). On the other hand, P concentration in leaves and stems increased in the two water conditions (AWD and PWD) in plants with Si fertigation in crops in the three tropical soils (Table 1).

### Stoichiometric ratios of C:N, C:P, C:Si, and N:P

**Fig. 1:**
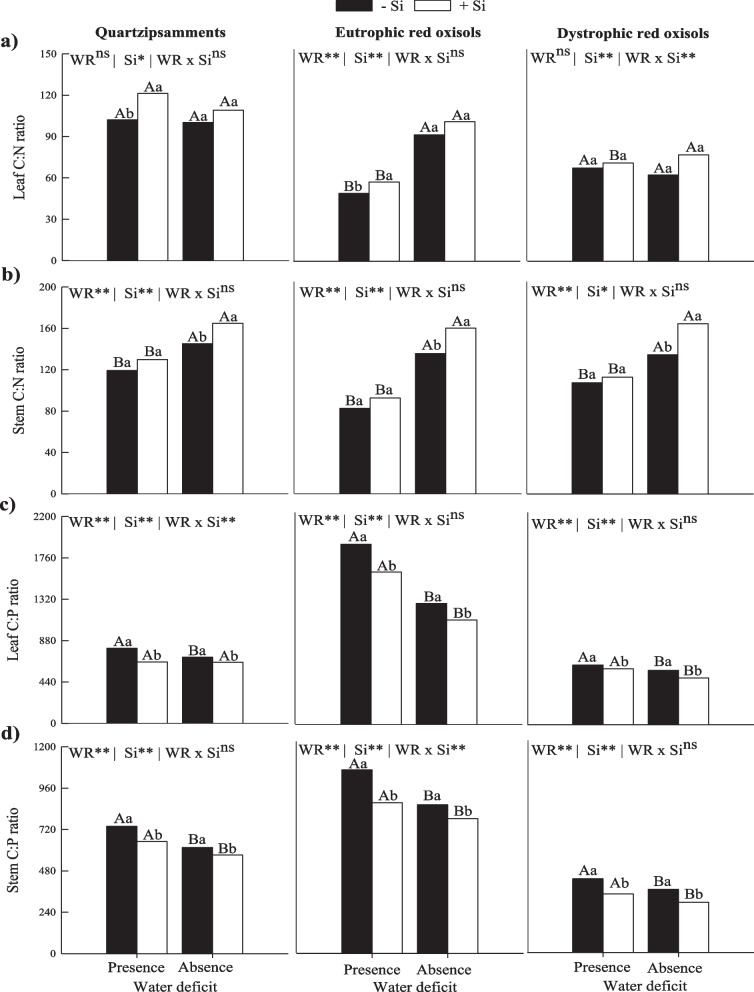
Stoichiometric ratios of C:N (**a**, **b**) and C:P (**c**, **d**) in leaves and stems of sugarcane plants in two water deficit conditions (presence: 35%, and absence: 70% of water retention) combined with absence (0.0 mmol L^−1^) and presence of fertigated Si (1.8 mmol L.^−1^) in three tropical soils (Quartzarenic Neosol; Eutroferric Red Latosol; and Dystrophic Red Latosol). WR: water retention capacity; ** and *: significant at 1% and 5% probability, respectively; NS = not significant at 5% probability. Different uppercase letters indicate differences in water deficit and different lowercase letters indicate differences in Si fertigation by Tukey test (*p* < 0.05)

Water deficit reduced the stoichiometric C:N ratio in leaves of sugarcane plants cultivated in Eutrophic Red Oxisols in the absence of Si application, and there was a similar decrease response in the C:N ratio in stems in all three soils of tropical regions (Fig. 1a and 1b). On the other hand, the application of Si via fertigation promoted an increase in the stoichiometric ratio C:N of leaves in PWD in Quartzipsamments and Eutrophic Red Oxisols, while it did not differ in Dystrophic Red Oxisols (Fig. 1a). For the stoichiometric ratio C:N in stems, there was also an increase promoted by Si fertigation in AWD (Fig. 1b).

The C:P ratio in leaves and stems increased in the presence of water deficit in sugarcane plants grown in the absence of Si fertigation in all three tropical soils (Fig. 1c and 1d). Furthermore, there was a decrease in the C:P ratio of leaves and stems of plants with Si fertigation in the two water conditions studied in all three tropical soils (Fig. 1c and 1d).

**Fig. 2:**
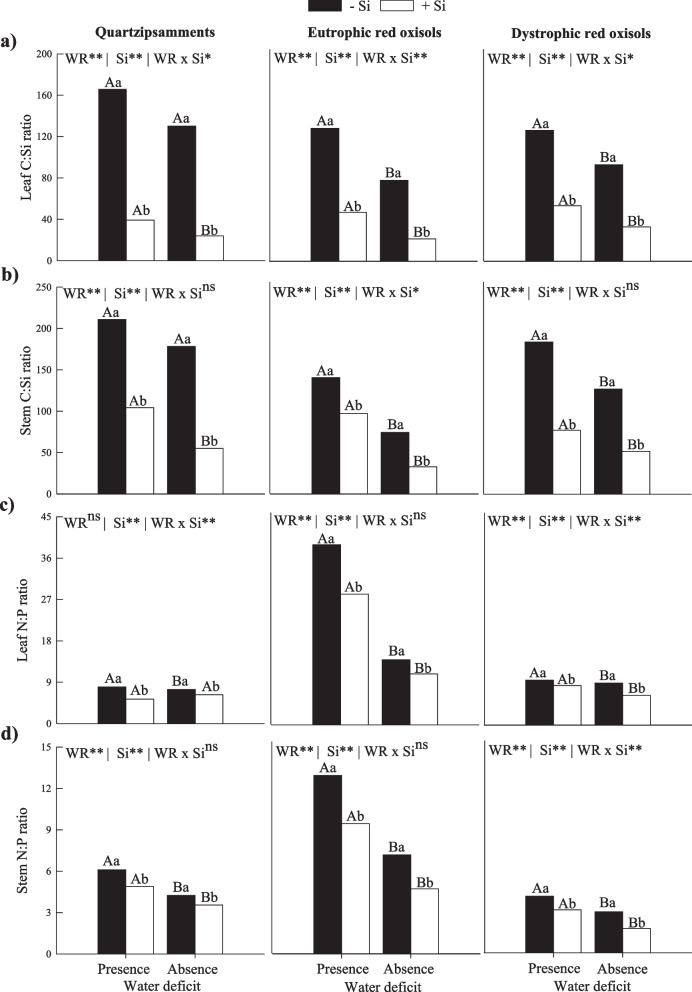
Stoichiometric ratios of C:Si (**a**, **b**) and N:P (**c**, **d**) in leaves and stems of sugarcane plants under two water deficit conditions (presence: 35%, and absence: 70% of water retention) combined with absence (0.0 mmol L^−1^) and presence of fertigated Si (1.8 mmol L.^−1^) in three tropical soils (Quartzarenic Neosol; Eutroferric Red Latosol; and Dystrophic Red Latosol). ** and *: significant at 1% and 5% probability, respectively; NS = not significant at 5% probability. Different uppercase letters indicate differences in water deficit and different lowercase letters indicate differences in Si fertigation by Tukey test (*p* < 0.05)

Water deficit also caused an increase in the C:Si ratio in leaves and stems in plants in the absence of fertigation with Si in Eutrophic Red Oxisols and Dystrophic Red Oxisols (Fig. 2a and 2b). On the other hand, there was a decrease in the C:Si stoichiometric ratio in leaves and stems of sugarcane fertigated with Si in the two water conditions studied here in the three tropical soils (Fig. 2a and 2b).

The stoichiometric ratio of N:P in leaves and stems also increased in the presence of water deficit in the absence of Si application in all three tropical soils (Fig. 2c and 2d). Meanwhile, there was a decrease in the stoichiometric ratio of N:P in leaves and stems in the presence of fertigation with Si in sugarcane plants in the two water conditions studied here in all three tropical soils (Fig. 2c and 2d).

### Si, C, N, and P contents

Water deficit reduced the Si content in leaves and stems in plants without Si application in the three tropical soils (Table 2). Meanwhile, fertigation with Si increased the Si content in leaves and stems of sugarcane in the two water conditions studied here in the three tropical soils (Table 2).

**Table 2:** C, N, P, and Si content in leaves and stem of sugarcane plants in two water deficit conditions (presence: 35%, and absence: 70% of water retention) combined with absence (0.0 mmol L^−1^) and presence of fertigated Si (1.8 mmol L^−1^) in three tropical soils (Quartzarenic Neosol; Eutroferric Red Latosol; and Dystrophic Red Latosol)

Water deficit	Si	Quartzipsamments		Eutrophic Red Oxisols		Dystrophic Red Oxisols	
		**leaf**	**Stem**	**leaf**	**Stem**	**leaf**	**Stem**
C (mg per plant)							
Presence	without	7013.7 Bb	1772.8 Bb	3783.3 Bb	514.4 Bb	8339.7 Bb	1850.2 Bb
	with	9722.6 Ba	2669.5 Ba	4431.1 Ba	1129.2 Ba	8909.7 Ba	2125.1 Ba
Absence	without	12,234.0 Aa	4577.5 Aa	5522.8 Ab	1571.7 Aa	11,377.0 Aa	3520.7 Ab
	with	12,443.0 Aa	5099.8 Aa	6644.0 Aa	1906.2 Aa	11,642.0 Aa	3865.6 Aa
N (mg per plant)							
Presence	without	69.87 Bb	15.72 Bb	77.14 Aa	6.24 Bb	122.66 Ba	17.72 Ba
	with	87.96 Ba	20.56 Ba	77.25 Aa	12.19 Aa	126.36 Ba	19.61 Ba
Absence	without	122.52 Aa	31.56 Aa	55.44 Ba	11.29 Aa	171.65 Aa	29.05 Aa
	with	119.00 Aa	30.31 Aa	63.82 Ba	11.70 Aa	161.20 Ab	23.89 Aa
P (mg per plant)							
Presence	without	8.78 Bb	2.59 Bb	2.00 Bb	0.49 Bb	13.17 Bb	4.33 Bb
	with	15.05 Ba	4.07 Ba	2.59 Ba	1.31 Ba	14.79 Ba	6.27 Ba
Absence	without	17.44 Ab	7.49 Aa	4.19 Ab	1.68 Ab	19.37 Ab	10.01 Ab
	with	19.28 Aa	8.91 Aa	6.07 Aa	2.51 Aa	23.34 Aa	13.26 Aa
Si (mg per plant)							
Presence	without	50.81 Bb	8.76 Bb	31.44 Bb	4.67 Bb	67.20 Bb	10.37 Bb
	with	242.51 Ba	24.87 Ba	95.53 Ba	12.43 Ba	168.06 Ba	28.58 Ba
Absence	without	96.79 Ab	28.60 Ab	66.94 Ab	21.55 Ab	144.02 Ab	26.93 Ab
	with	534.00 Aa	94.40 Aa	299.06 Aa	63.01 Aa	346.45 Aa	79.48 Aa

Different uppercase letters indicate differences in water deficit and different lowercase letters indicate differences in Si fertigation by Tukey test (*p* < 0.05)

C content decreased in leaves and stems in plants under PWD, irrespective of Si supply. in different tropical soils (Table 2). On the other hand, fertigation with Si increased the C content in leaves and stems of plants in the two water conditions in all three tropical soils. However, plants grown in Quartzipsamments and Dystrophic Red Oxisols showed an increase in C content in leaves only under PWD and in stems only under PWD in Quartzipsamments and Eutrophic Red Oxisols (Table 2).

In the absence of Si supply, water deficit reduced N contents in leaves and stems in sugarcane cultivated in Quartzipsamments and in Dystrophic Red Oxisols, while in Eutrophic Red Oxisols the N content was reduced only in the stem (Table 2). On the other hand, the N content in leaves of sugarcane fertigated with Si increased in PWD in Quartzipsamments and in AWD in Dystrophic Red Oxisols but it did not differ in Eutrophic Red Oxisols (Table 2). For stems, there was an increase in N content in sugarcane fertigated with Si under PWD in Quartzipsamments and Eutrophic Red Oxisols, however, there was no response in Dystrophic Red Oxisols (Table 2).

Water deficit caused a reduction of P content in leaves and stems in sugarcane plants with and without Si application in all three tropical soils (Table 2). The supply of Si increased P content in sugarcane leaves in the two water conditions (AWD and PWD) in the three tropical soils (Table 2). On the other hand, the P content in stems increased with Si fertigation in both water conditions (AWD and PWD); however, in Quartzipsamments there was an increase only in PWD (Table 2).

### C, N, and P use efficiency and biomass partition

**Fig. 3:**
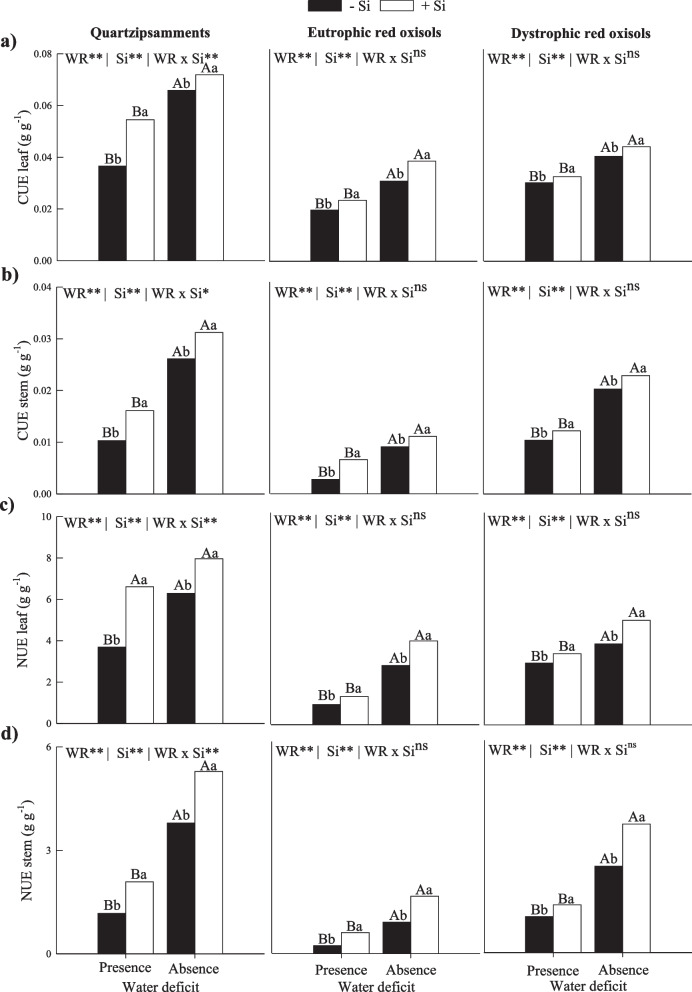
Efficiency use of carbon (**a**, **b**) and nitrogen (**c**, **d**) in leaves and stems of sugarcane plants in two water deficit conditions (presence: 35%, and absence: 70% of water retention) combined with absence (0.0 mmol L^−1^) and presence of fertigated Si (1.8 mmol L.^−1^) in three tropical soils (Quartzarenic Neosol; Eutroferric Red Latosol; and Dystrophic Red Latosol). ** and *: significant at 1% and 5% probability, respectively; NS = not significant at 5% probability. Different uppercase letters indicate differences in water deficit and different lowercase letters indicate differences in Si fertigation by Tukey test (*p* < 0.05)

The reduction in C use efficiency in leaves and stems of plants occurred in the absence of Si fertigation in all three tropical soils (Fig. 3a and 3b). The application of Si via fertigation increased the efficiency of C use in leaves and stems of plants in the two water conditions studied here in the three soils (Fig. 3a and 3b).

Water deficit also induced a reduction in N use efficiency in leaves and stems in plants without Si fertigation in the three tropical soils (Fig. 3c and 3d). This increase in efficiency also occurred for N use efficiency in leaves and stems in sugarcane fertigated with Si in the two water conditions studied in the three tropical soils (Fig. 3c and 3d).

**Fig. 4:**
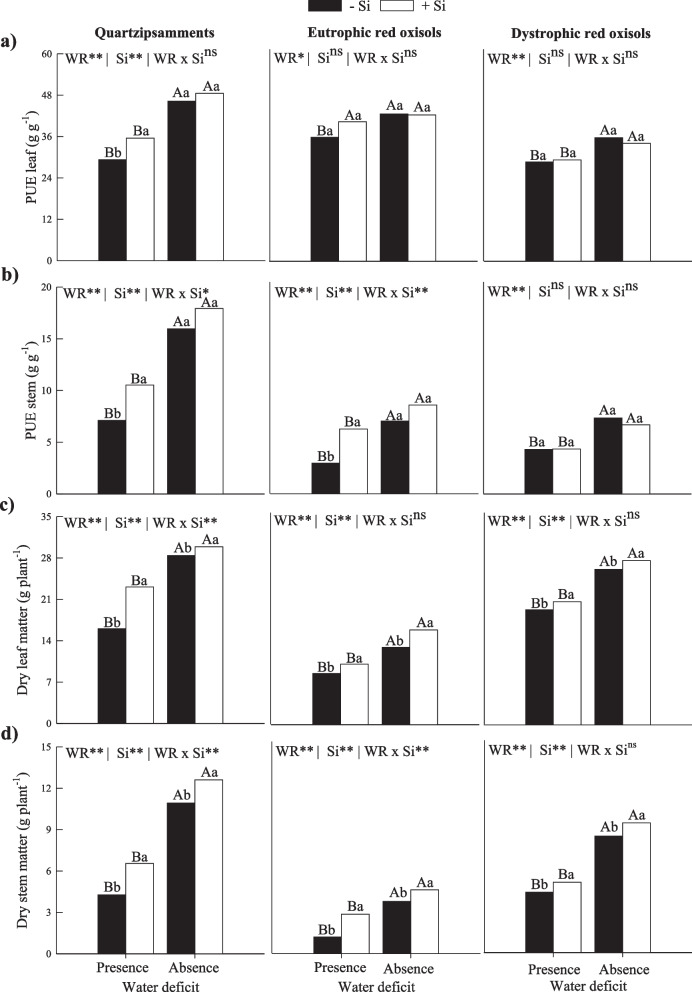
Phosphorus use efficiencies (**a**, **b**) and dry mass partition (**c**, **d**) in leaves and stems of sugarcane plants in two water deficit conditions (presence: 35%, and absence: 70% of water retention) combined with absence (0.0 mmol L^−1^) and presence of fertigated Si (1.8 mmol L.^−1^) in three tropical soils (Quartzarenic Neosol; Eutroferric Red Latosol; and Dystrophic Red Latosol). ** and *: significant at 1% and 5% probability, respectively; NS = not significant at 5% probability. Different uppercase letters indicate differences in water deficit and different lowercase letters indicate differences in Si fertigation by Tukey test (*p* < 0.05)

The efficiency of P use in leaves and stems also decreased in the PWD in sugarcane without Si fertigation in different tropical soils (Fig. 4a and 4b). In PWD, there was an increase in the efficiency of P use in leaves in sugarcane fertigated with Si in Quartzipsamments. However, there was no difference in Eutrophic Red Oxisols and Dystrophic Red Oxisols (Fig. 4a). For stems, there was also an increase in the efficiency of P use in sugarcane fertigated with Si in PWD in Quartzipsamments and Eutrophic Red Oxisols; however, there were no responses in plants grown in Dystrophic Red Oxisols (Fig. 4b).

Water deficit caused a reduction in the production of dry mass in leaves and stems in plants without Si application in all three tropical soils. On the other hand, the production of dry mass by leaves and stems increased with Si fertigation in the two water conditions studied here in the three tropical soils (Fig. 4c and 4d).

### Multivariate analyses

#### Hierarchical cluster analysis

**Fig. 5:**
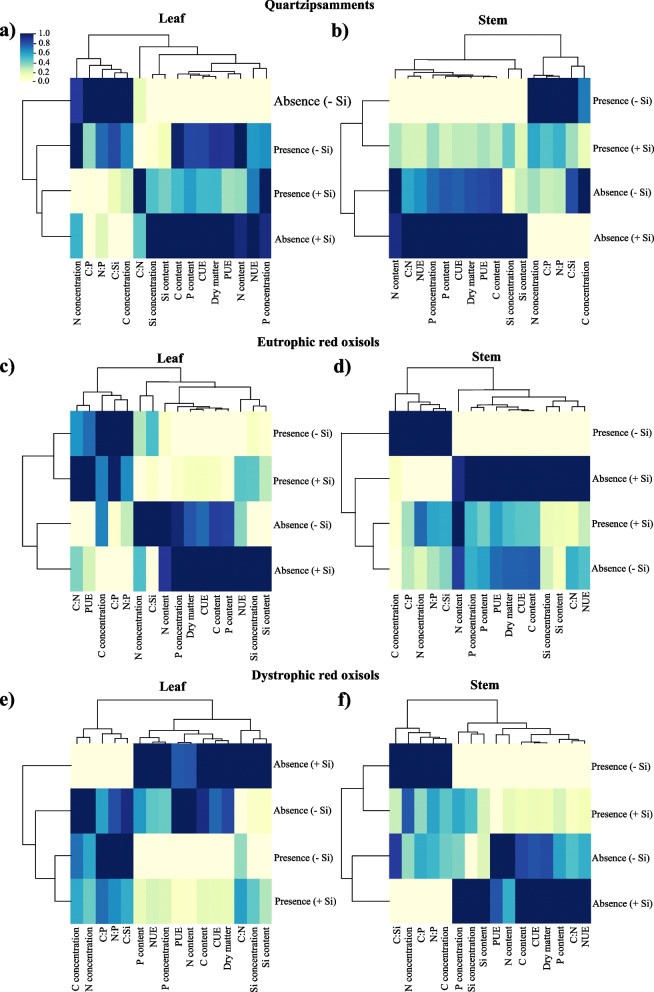
Heat map of hierarchical clustering of variables of concentrations and accumulations of C, N, P, and Si, stoichiometric ratios of C:N:P:Si, use efficiency of C, N, and P and dry mass partition in leaves (**a**, **c**, **e**) and stems (**b**, **d**, **f**) of sugarcane plants in two water deficit conditions (presence: 35%, and absence: 70% of water retention) combined with absence (0.0 mmol L^−1^) and presence of fertigated Si (1.8 mmol L^−1^) in three tropical soils (Quartzarenic Neosol; Eutroferric Red Latosol; and Dystrophic Red Latosol). CUE: C use efficiency; NUE: N use efficiency; PUE: P use efficiency

The hierarchical cluster analysis of leaves indicated that under PWD conditions without Si fertigation there was a greater dissimilarity from the other conditions evaluated in Quartzipsamments, while in Dystrophic Red Oxisols there was a greater dissimilarity in AWD with Si fertigation and in Eutrophic Red Oxisols. Two groups were formed related to the two water conditions (Fig. 5). For response variables, there were similar responses for the three soils as for leaves and stems, indicating the formation of two groups, the first group formed by concentrations of C and N and the stoichiometric ratios C:P, C:Si, and N:P, and the second group formed by the concentrations of P and Si, stoichiometric ratio C:N, contents of C, N, P, and Si, efficiency of use of C, N, and P and dry mass. However, the efficiency of P use in leaves was found for the first group rather than for the second group in Eutrophic Red Oxisols (Fig. 5).

In Quartzipsamments, cluster analysis indicated an association of similarity of Si and P concentrations, C, P, and Si contents, C, N, and P use efficiencies, and dry mass in AWD fertigated with Si in leaves and stems, while the stoichiometric ratios of N:P, C:P, and C:Si and the concentration of C were associated with PWD without Si fertigation in leaves and stems (Fig. 5a and 5b). Furthermore, N concentration and content were associated with AWD without Si fertigation in leaves and stems (Fig. 5a and 5b).

For Eutrophic Red Oxisols, the cluster analysis indicated a greater similarity of P and Si concentrations, C, P, and Si contents, C and N efficiencies, and dry mass to AWD fertigated with Si in leaves (Fig. 5c), while in stems there was a greater association of P and Si concentrations, C, P, and Si contents, C, N, and P use efficiencies, C:N stoichiometric ratio, and dry mass to AWD fertigated with Si (Fig. 5d). For PWD without Si fertigation, there was a greater association of C concentration and C:P and N:P stoichiometric ratios in leaves and C and N concentrations, C:P stoichiometric ratios, and C:Si and N:P in stems (Fig. 5c and 5d). Furthermore, the P use efficiency and the C:N ratio were associated with PWD fertigated with Si and the concentration, and the content of N and C:Si ratio were associated with AWD without Si fertigation in leaves (Fig. 5c).

In AWD fertigated with Si in Dystrophic Red Oxisols, there was an association with P and Si concentrations, C, P, and Si contents, C and N use efficiencies, C:N stoichiometric ratio, and dry mass in leaves and stems (Fig. 5e and 5f). For AWD without Si fertigation, there was an association between N and C concentrations, N content, and P use efficiency in leaves, while in stems there was an association between P use efficiency and N content (Fig. 5e and 5f). Finally, for PWD without Si fertigation, the stoichiometric ratios C:P, C:Si, and C:Si in leaves and the concentrations of C and N and the stoichiometric ratios C:P, C:Si, and C were associated with Si in stems (Fig. 5e and 5f).

#### Principal component analysis

**Fig. 6:**
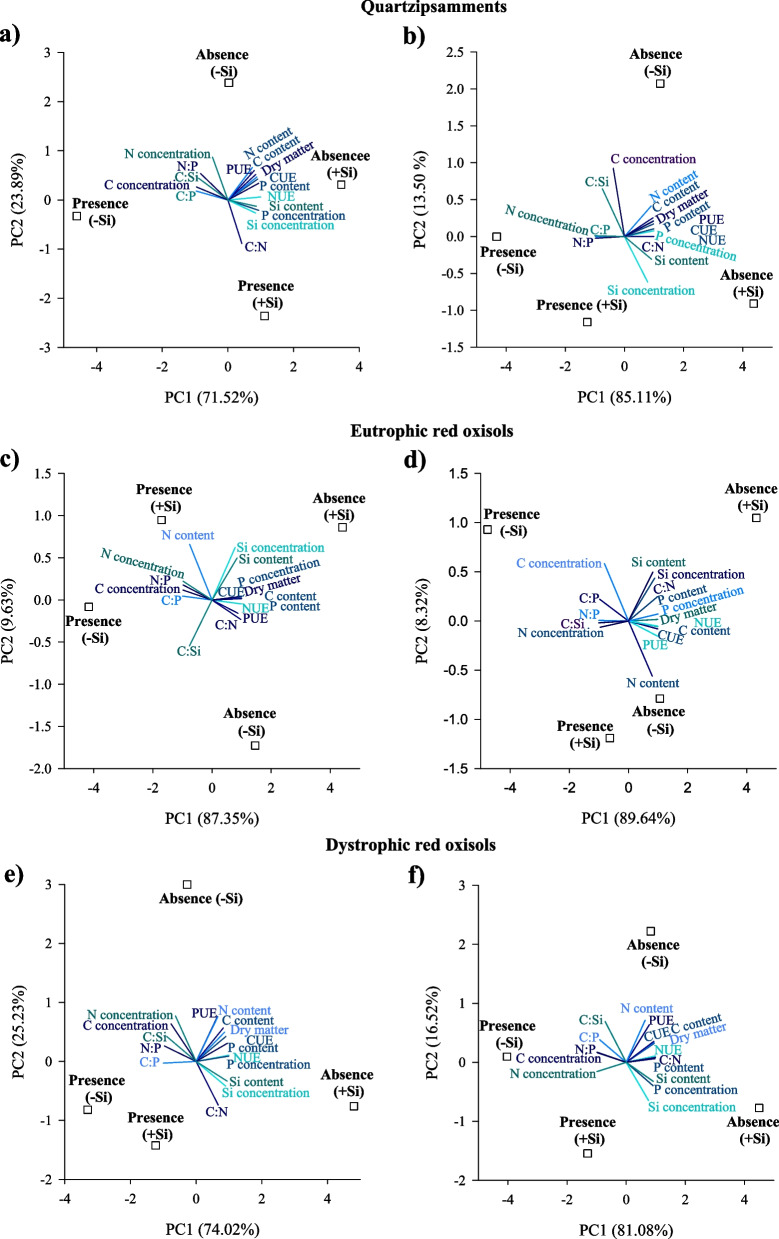
Principal component analysis of variables related to concentrations and accumulations of C, N, P, and Si, stoichiometric ratios of C:N:P:Si, use efficiency of C, N, and P and dry mass partition in leaves (**a**, **c**, **e**) and stems (**b**, **d**, **f**) of sugarcane plants in two water deficit conditions (presence: 35%, and deficit: 70% of water retention) combined with absence (0.0 mmol L^−1^) and presence of fertigated Si (1.8 mmol L^−1^) in three tropical soils (Quartzarenic Neosol; Eutroferric Red Latosol; and Dystrophic Red Latosol). CUE: C use efficiency; NUE: N use efficiency; PUE: P use efficiency

Principal component analyses (PCA) of leaves explained 95.4, 97.0, and 99.3% of the variable responses of sugarcane cultivated in Quartzipsamments, Eutrophic Red Oxisols, and Dystrophic Red Oxisols, respectively (Fig. 6). For stems, the PCA explained 98.6, 98.0, and 97.6% in cultivation on Quartzipsamments, Eutrophic Red Oxisols, and Dystrophic Red Oxisols (Fig. 6).

In Quartzipsamments, the PCA of leaves indicated an association of C and N concentrations and stoichiometric ratios C:P, C:Si, and N:P to AWD and PWD without Si fertigation and C and N contents to AWD with absence and presence of Si fertigation, while P content, C and N use efficiency, and dry mass production associated with Si fertigation in AWD (Fig. 6a). Also, P and Si concentrations, Si content, and dry C:N ratio associated with Si fertigation in both water conditions (Fig. 6a). For stem, the PCA indicated an association of C and N concentrations to AWD and PWD without Si fertigation and the stoichiometric ratios C:P, C:Si, and N:P to PWD without Si fertigation. The stoichiometric ratios C:P, C:Si, and N:P, Si concentration and content, and C:N ratio associated with AWD and PWD with Si fertigation (Fig. 6b). Furthermore, C, N, and P contents, C, N and P use efficiencies, P concentration, C:N ratio, and dry mass associated with AWD with the absence and presence of fertigation with Si (Fig. 6b).

For Eutrophic Red Oxisols, regarding the response variables in leaves, C and N concentrations and the N:P ratio associated with PWD with the absence and presence of fertigation with Si, and the N content associated with PWD fertigated with Si, while the stoichiometric ratio C:Si associated with the two water conditions with the absence of fertigation with Si (Fig. 6c). It also showed that the highest concentration and content of Si associated with AWD fertigated with Si, and that the C:P ratio associated with PWD without Si fertigation (Fig. 6c). Additionally, P concentration, C and P contents, C, N, and P use efficiency, C:N ratio, and dry mass production associated with AWD with the absence and presence of Si fertigation (Fig. 6c). In stems, the PCA showed association of C:P, N:P, and C:P ratios and N concentration to PWD with the absence and presence of fertigation with Si, and the concentration of Si, P and Si contents, and the C:N ratio associated in AWD with Si fertigation (Fig. 6d). Also, the PCA showed that the highest C concentration associated with PWD without fertigation with Si, and the highest N content associated with AWD fertigated with Si (Fig. 6d). Furthermore, P concentration, C content, C, N, and P use efficiency, and dry mass associated with AWD with the absence and presence of fertigation with Si (Fig. 6d).

For the PCA of response variables in leaves of sugarcane cultivated in Dystrophic Red Oxisols, there was an association between the concentrations of N and C and the stoichiometric ratios of C:P, C:Si, and N:P to PWD and AWD in the absence of Si fertigation. Also, the concentration and content of Si and the C:N ratio also associated with the two water conditions, however with the presence of Si fertigation (Fig. 6e). It also showed that C, N, and P use efficiencies, C, N, and P contents, P concentration, and dry mass production associated with AWD in the absence and presence of Si fertigation (Fig. 6e). The PCA of stem response variables indicated an association of the stoichiometric ratios N:P, C:P, C:Si and the concentration of C to PWD in the absence of fertigation with Si; it also indicated an association of the concentration of N to PWD in the absence and presence of Si fertigation (Fig. 6f). The PCA of stem response variables also evidenced an association of P and Si concentrations and Si content to AWD in the presence of Si fertigation, and the association of N content and P use efficiency associated to AWD in the absence of Si fertigation (Fig. 6f). Moreover, C and N use efficiency, C and P contents, and other above dry mass also associated with AWD in the absence and presence of Si fertigation (Fig. 6f).

## Discussion

### Biological damages of water deficit to sugarcane ratoon without Si supply

Losses by water deficit in sugarcane crops have already been related to the increase in water loss in tissues, which induces oxidative stress and decreases photosynthetic rates [11]. However, recent studies have shown nutritional damage especially in the homeostasis of nutrients with a vital structural function for plant growth [13, 14].

In this research, we showed that water deficit changes the homeostatic balance of C:N:P, which occurs due to the decrease in the C:N ratio in stems and the increase in the stoichiometric ratios C:P and N:P in leaves and in stems in three tropical soils (Fig. 1 and 2). Additionally, water deficit reduced the stoichiometric ratio C:N in leaves of sugarcane plants cultivated in Eutrophic Red Oxisols and decreased the stoichiometric ratio C:Si in sugarcane plants cultivated in Eutrophic Red Oxisols and Dystrophic Red Oxisols. In regrowth of *Panicum maximum*, the stoichiometric ratio C:N decreased, reducing the concentration of C and causing instability in the metabolism of the culture [26]. However, plants of the first cycle of *Panicum maximum* had no impact of water deficit on C:N stoichiometry ratio, as observed for the cultivar Masai. However, the C:N ratio changed in the BRS Zuri cultivar [27]. These findings show that impacts of the water deficit can change depending on the cultivar in the first cycle; while this tolerance is lost in the second cycle (stubs or regrowth). Our results reinforce that the deficit impacts are more severe in stumps, directly impacting C:N:P homeostasis.

These alterations in the homeostatic balance of C:N:P caused by water deficit result in direct implications to the concentrations of these elements, causing a clear decrease in the C, N, and P contents of the plant (Tables 1 and 2). This decrease in N and P absorption in most of the studied soils (Table 2) and a deficient water regime possibly happens due to the mobility of nutrients in the soil, which reduces their diffusion until reaching the surface of roots and their entry into the plant [28]. Water deficit also induces a reduction in the transport of nutrients from plant roots to shoots due to a decrease in the transpiration rate, which changes the efficiency of transporters [29]. In addition, water deficit, by decreasing the redistribution of nutrients in plants, impairs the internal cycling of nutrients [30], aggravating the homeostatic balance C:N:P, and causes direct effects on the reduction in the use efficiency of C, N, and P (Fig. 3 and 4). This results in losses of dry mass in leaves and in stems in all three tropical soils studied (Fig. 4c and 4d).

Therefore, our results prove that the disturbances caused by water deficit in relation to the plant that receives an adequate irrigation have a strong nutritional component that causes an imbalance of C:N:P, impairing nutritional efficiency and consequently contributing to productive loss of sugarcane crops. It is quite clear that the loss of elementary stoichiometric homeostasis of sugarcane caused by water deficit paves the way for future specific studies on proteomics involving the cycling of the nutrients C, N, and P seeking to better understand the underlying effects of water stress on this species.

### Biological contribution of Si in sugarcane ratoon with deficient irrigation

Studies with application of Si via fertigation in sugarcane crops with deficient irrigation are restricted [13, 14, 31–33]. This raises concerns, as it may decrease the absorption of Si depending on the soil and restrict its biological benefits in the plant.

This problem did not occur in this study, as we showed that fertigation with Si in sugarcane plants, in relation to its absence, is efficient in increasing the concentration and content of Si in leaves and stems in all three tropical soils even with irrigation deficit (Tables 1 and 2). This is because sugarcane plants have efficient Si transporters [34].

Such increases in Si content in sugarcane plants due to fertigation with Si in relation to the absence of Si application are sufficient to reduce the nutritional imbalance water deficit causes. This occurs because the use of Si increases the C:N stoichiometric ratio in leaves of plants grown in Quartzipsamments and Eutrophic Red Oxisols and decreases the C:P, C:Si, and N:P ratio in leaves and stems in all three tropical soils (Fig. 1 and 2). These changes in C:P:N stoichiometry induce a new homeostatic equilibrium, tending to reestablish stoichiometric relationships similar as those of sugarcane plants grown in an adequate water regime.

The induction of a new homeostatic balance of C:N:P caused by Si under water deficit conditions also occurs in 30-day pre-sprouted seedlings [13], and 90-days [32], 150-days [33] and 160-days [31] sugarcane plants. The induction of a new C:N:P homeostasis is not only restricted in sugarcane plants, but has also been observed in forage [26, 27], quinoa [35], wheat [36], sorghum and sunflower [17, 37]. This helps to consolidate another benefit of Si in the cultivation of sugarcane that also occurs in sugarcane ratoons, but it is not restricted only to this crop. At this crop stage, little is known about the effects of homeostatic changes in C:N:P in sugarcane and mainly its implications to the production of biomass in stalks in different tropical soils. In this scenario, stoichiometric modifications change ecological interactions due to stress, but the supply of Si is effective to contribute to reverse this stress, maintaining optimized biological and biochemical functions of nutrients in agroecosystems [38].

This stoichiometric homeostasis caused by Si generated another benefit to the supply of Si in sugarcane crops with water deficit, as it increased the contents of C, N, and P in sugarcane plants cultivated in all three tropical soils (Table 2). Si positively affects N and P nutrition, increasing the efficiency of NH_4_^+^ transporters (OsAMT), stimulating NO_3_^−^ expression (BnaNTR2.1), increasing gene expression related to inorganic phosphorus (Pi) uptake and upregulating the gene coding of Pi transporters (TaPHT1;1 and TaPHT1;2) [39]. Furthermore, there are several reports of increased concentrations of this nutrient in the presence of Si under low N availability, indicating the synergistic effect of the two elements [40–44]. In quinoa, Si's role in controlling the C flux modified N and P concentrations by increasing the concentrations of theoe nutrients [35]. Moreover, this homeostasis promoted by Si also increased the use efficiency of these nutrients in leaves and stems in the three soils studied, except for P in Dystrophic Red Oxisols (Fig. 3). Thus, it is possible that the stoichiometric change, such as the decrease in the C/Si ratio, as mentioned above, due to the absorption of Si by the crop under deficient irrigation, may contribute to explain the increase in the use efficiency of C. This is because, according to Kim et al. (2007)[45], plants can employ strategies of using Si to replace C in the cell wall, providing lignin-like structural strength.

The biological role of Si in replacing C in cell walls also contributes to altering N and P metabolism, as there is an improvement in the photosynthetic apparatus. This increases the photosynthetic rate of plants, decreases the C concentration and increases the N and P concentrations [22]. Possibly, there is a multiple effect of Si on N metabolism, but not yet fully elucidated. New studies to understand the mechanisms of Si in the modification of N metabolism are still necessary, mainly on the implication of Si in the homeostasis of nutrients. So far, the proportions of modification of this beneficial element in nutrient metabolism and its implications for the sustainability of agricultural systems are unknown.The beneficial interaction of P and Si is possible to occur in two ways resulting in the improvement of P metabolism, being the improvement of the efficiency of absorption and the increase of efficiency of use by tissues [39]. In fact, several reports are found in the literature on stressed plants about the beneficial effect of Si in increasing the P concentration [33, 46]. Evidence indicated that Si improves the absorption of P by increasing the biosynthesis of exudates, such as malate and citrate, which have the role of competing with P for adsorption sites or, even, forming complexes of Al and Fe, increasing the availability of P in the soil solution [46, 47].

The results indicate the importance of C, N, and P homeostasis in reaching a stabilization to optimize the metabolism of these nutrients and, consequently, increase the capacity of plants to convert nutrients into biomass [21]. This fact was confirmed here since the using Si in plants under water deficit promoted an increase in the biomass content of leaves and stems in all three soils studied.

Thus, it is evident that Si can attenuate the water deficit in sugarcane in the studied soils. However, as multivariate analysis showed, it was possible to detect additional information showing that the level of such mitigation was more intense in one type of soil than in the others (Fig. 5). The response in terms of stem production of the crop grown under water deficit that received Si was similar as that of plants with an adequate irrigation (in the absence of Si), but this only occurred in Eutrophic Red Oxisols. In other words, in the other soils, Si did not induce such a high performance in plants. This result highlights the agronomic importance of using Si in sugarcane cultivation in Eutrophic Red Oxisols, indicating that it is possible to completely reverse the decreases in productivity by cultivating with irrigation deficit by equaling an adequate irrigation. This indicates that the efficiency of silicate fertilization is influenced by soil type, showing the importance of considering the mineralogical composition, texture, contents of Fe and Al oxides, and soil organic matter [25], in addition to the levels of available Si in the soil. The intensity of the beneficial effects of Si under water deficit changes according to edaphic characteristics. Consequently, the intensity of modification of the homeostatic balance C:N:P increased, especially the C:N ratio of 9, 18, and 4% for Quartzipsamments, Eutrophic Red Oxisols, and Dystrophic Red Oxisols, respectively (Fig. 1 and 2). This greater intensity of modification of this stoichiometric ratio in Eutrophic Red Oxisols may have contributed to the greater benefit of Si in attenuating the deleterious effects of water deficit.

It was clear that our results are sufficient to confirm the second hypothesis, i.e., the results indicate that the using Si can modify the stoichiometry of C:N:P and mitigate the damage caused by water deficit by improving the nutritional efficiency of these nutrients, which in turn affects the productivity of sugarcane ratoon cultivated with deficient irrigation in the three soils studied. It is possible that the impact of Si on nutritional stoichiometry is driven by two paths, the first being the increase in the efficiency of nutrient use and, the second, the activation of physiological mechanisms that increase the efficiency of nutrient absorption; however, this needs to be proven.

Therefore, this research shows concrete indications that contribute to the sustainability of sugarcane cultivation in irrigated systems with water deficit by using Si, exerting an environmental implication due to the decrease in water use. This is important, as the water demand for irrigated systems is expected to increase in the coming years [2], in addition to the costs, especially of electric energy [5].

### Biological contribution of Si in improving the performance of sugarcane ratoon with adequate irrigation

Fertigation with Si in an adequate water regime, in relation to its absence, improved Si absorption and increased the concentration and contents of Si in leaves and stems of sugarcane plants cultivated in the three tropical soils (Tables 1 and 2). The greater absorption of Si by sugarcane plants occurs because of a greater transpiration. Consequently, the mass flow of nutrients increased due to a greater availability of water in the soil [48]. The higher water supply to plants that received Si in relation to its absence allowed increasing the Si content in leaves by 5.5, 4.5, and 3.0 times in plants grown in Quartzipsamments, Eutrophic Red Oxisols, and Dystrophic Red Oxisols, respectively (Table 2). This indicates a greater recovery of Si applied to plants grown in Quartzipsamments in relation to the other soils possibly due to the lower adsorption of anionic Si in this soil, although Si may be more mobile in this soil, especially in water regimes more voluminous than the one studied, that is, 70% of the water retention capacity.

The greater efficiency of Si fertigation resulted in the alteration of the homeostatic balance C:N:P, which was evidenced with the increase in the C:N ratio only in stems, which in turn indicates a lower demand of C per unit of N, i.e., a preliminarily better use of N in the metabolism of stress-free plants. In addition, the use of Si caused a decrease in C:P, C:Si, and N:P ratios in leaves and stems of sugarcane plants cultivated with adequate irrigation in the three tropical soils. This may indicate a greater demand of metabolism of P and Si (Fig. 1 and 2).

Therefore, the results prove these beneficial effects of Si in sugarcane plants with adequate irrigation because causing a homeostasis of C:N:P directly promotes an increase in the use efficiency of these nutrients. The effects of a greater efficiency in the use of N and P contribute to increase the performance of their metabolic functions that are involved with the composition of vital organic compounds for physiological processes [21], thus contributing to increase the use efficiency of C. Changes in the primary metabolism of plants caused by Si may induce a greater redistribution of amino acids to the draining tissues and increase the use efficiency of N [49, 50] and the use efficiency of P [13].

In addition, noteworthy was an increase in the use efficiency of C also associated with a decrease in the stoichiometric ratio C:Si. This may occur because there are reports that Si can replace C in organic compounds with low metabolic costs if compared to that of C, generating ten to 20 times less metabolic energy for incorporation into organic molecules [51]. In plants grown under stress, this can also occur, as indicated above, but in plants without stress there is a tendency for this energy balance to be directed towards the synthesis of biomass and consequently in crop productivity. This is due to the optimization of C metabolism, which increases the production of organic compounds, including C skeletons and structural nutrients such as N and P [52]. Therefore, the findings prove the importance of Si in the nutrition of stress-free sugarcane plants grown in different tropical soils. This reinforces the finding that Si is important not only to plants under stress, but also to plants under no stress [15, 22]. Our research contributes to the thesis that this occurs due to Si role in C, N, and P homeostasis, which is little reported in the literature.

The better performance of plants induced by fertigation with Si and by favoring nutritional efficiency of C, N, and P directly reflects on the synthesis of biomass of leaves and stems, as observed in the three tropical soils studied here (Fig. 4c and 4d). The PCA analysis reinforces that dry mass biosynthesis is completely related to increased C use efficiency in the three tropical soils cultivated with adequate irrigation (Fig. 6).

The benefit of Si in improving nutrient uptake and use efficiency was also evidenced in sorghum and sunflower plants, resulting in improved nutritional efficiency [53]. In wheat plants, it evidenced the role of Si in promoting the improvement of P nutrition, suggesting a possible allocation of P to the reproductive organs in detriment of the vegetative organs, consequently, increasing grain production [36], and the efficiency of P use. This is due to Si replacing parts of the C compounds [54] that allows allocation of energy resources.

The results of this research allow us to accept the third hypothesis, since the use of Si in sugarcane plants cultivated using adequate irrigation by increasing the element content in the plant is enough to modify the stoichiometric ratio C:N:P, thus increasing the efficiency of metabolism in increasing the use efficiency of C, N, and P and consequently the production of sugarcane biomass.

In general, deficient irrigation with no Si supply causes biological damage to sugarcane ratoons because Si can induce the loss of stoichiometric C:N:P homeostasis, which is responsible for reducing the use efficiency of these nutrients in sugarcane ratoon cultivated in all three tropical soils. However, it is possible to cultivate sugarcane under deficient irrigation because the use of fertigation with Si can mitigate losses, as it stabilizes the stoichiometric ratio C:N:P in a way that increases the efficiency of nutrient use and sustains the production of dry mass of sugarcane ratoon. However, the type of cultivation soil affects the intensity of Si benefits. In addition, these Si benefits repeated in plants with adequate irrigation, indicating that the effects of Si also happen in non-stressed plants.

Our finding, i.e., identifying that Si modulates the stoichiometric homeostasis of C:N:P, which directly interferes with the nutritional efficiency of these nutrients in sugarcane ratoon cultivated in different tropical soils, opens new paths for a sustainable cultivation of sugarcane under different water regimes. Therefore, this finding should greatly increase the use of Si in sugarcane crops, producing global implications, as it will be useful in many regions with a regular precipitation or irrigated areas and in regions with irregular precipitation or irrigated areas with low water availability.

## Methods

**Fig. 7:**
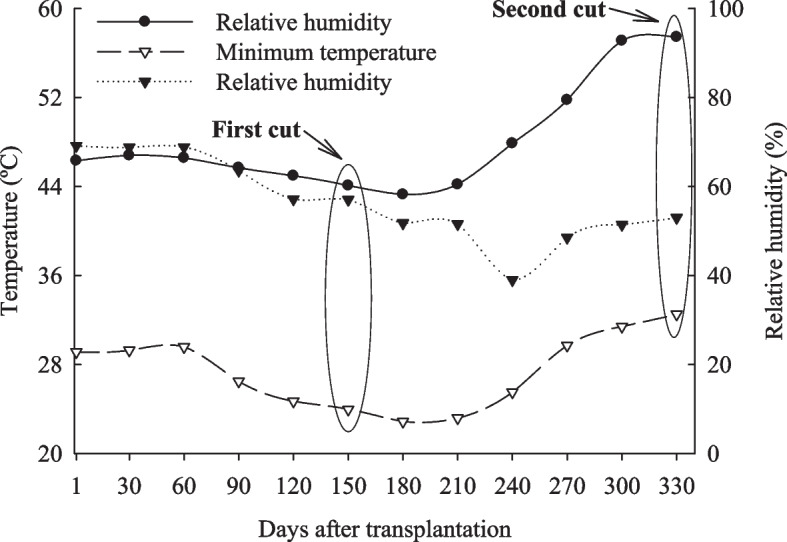
Air temperature and relative humidity during the experimental period

Three experiments were carried out in a greenhouse at São Paulo State University Júlio Mesquita Filho (UNESP), Jaboticabal (Brazil), using the variety RB 962,860 of sugarcane in three tropical soils: Quartzipsamments (NQ), Eutrophic Red Oxisol (LVe), and Dystrophic Red Oxisol (LVd). During the conduction of the study, meteorological data, maximum temperature (48.4 ± 5.1 ºC), minimum temperature (27.4 ± 3.4 ºC), and relative air humidity (55.7 ± 9.2%) at the site of the experiments were monitored daily using a thermo-hygrometer (Fig. 7).

The treatments were arranged in a 2 × 2 factorial design in randomized blocks. There were three experiments with two water conditions, with deficient and adequate irrigation corresponding to 35 and 70% of the soil water retention capacity, combined with the absence (0.0 mmol L^−1^) and the presence of Si (1.8 mmol L^−1^) applied via fertigation in five replications. The experimental plot was characterized by pre-sprouted sugarcane seedlings at 60 days of age, after sprout emergence, in polypyrene pots with a volume of 20 L, filled with 18 L of soil sample. Sugarcane seedlings of the RB 962,869 variety were purchased from a certified commercial plant nursery. The pre-sprouted seedlings, during production and throughout their growth, did not receive Si application. They were inoculated with *Rhyzobium meliloti* and, after transplanting, plants were pruned at 0.3 ± 0.02 m from the soil surface. The experiment was carried out in two cultivation cycles (plant cane and ratoon cane), of 150 days each. At the end of the first cultivation cycle, the plants were cut at 0.1 m from the soil surface; later, in the second cycle of cultivation, they were cut again after the new sprouting of plants (15 days after the cut).

Soil chemical analysis was performed before transplantation to characterize the chemical attributes [55], particle size [56], and determination of the Si concentration [57] (Table 3). Soil texture was characterized as sandy, loamy, and sandy loamy in Quartzipsamments, Eutrophic Red Oxisol, and Dystrophic Red Oxisol, respectively. Subsequently, liming was performed 45 days before transplantation using the base saturation method (V%), seeking to correct the saturation to 60% [55]. After 30 days of soil incubation, phosphate fertilization was carried out; during transplantation, nitrogen, potassium, and micronutrient fertilization were applied through fertigation, with N, K, and micronutrient fertilization in the second cycle after cutting the stems of the first crop cycle. Cover N and K fertilization were also performed 76 days after transplantation in the first cycle and 76 days after cutting the stems in the second cultivation cycle.

**Table 3:** Chemical characteristics of Quartzipsamments (NQ), Eutroferric Red Oxisols (LVe) and Dystrophic Red Oxisols (LVd)

Soil	pH	OM	P	S	K	Ca	Mg	Al	H + Al	CEC	V	m	Si	clay	silt	sand
	CaCl_2_	g dm^−3^	mg dm^−3^		––––– mmolc dm^−3^ –––––						%		mg kg^−1^	g kg^−1^		
NQ	4.3	9	2	6	0.3	3	1	0	16	20.0	21	0	1	50	10	940
Lve	6.2	8	8	8	1.0	16	5	0	17	38.6	57	0	5	550	240	210
LVd	5.2	9	20	7	1.2	14	6	0	22	44.2	49	0	3	300	40	660

*CEC* Cation exchange capacity, *V* base saturation, *m* aluminum saturation, *pH* CaCl_2_ by potentiometry, *H* + *Al* SMP buffer by potentiometry, *O.M*. organic matter by spectrophotometry, *P* in resin by spectrophotometry, *S* by turbidimetry, *K* Ca and *Mg* atomic absorption spectrometry, *Si* 0.01 M calcium chloride

To determine the soil water retention capacity, lysimeters (20-L pots) were used with a soil sample (18 L) in three replications for each experiment and placed in a 250-L water tank filled with water up to 2/3 of the height of lysimeters for a period of 24 h. The surface of lysimeters were insulated with plastic film. After a period of 24 h, the lysimeters were freely drained, and their masses were evaluated at 0, 24, 36, 48, 60, and 72 h to determine water replacement capacity by the difference in wet and dry soil masses. Subsequently, the gravimetric and volumetric moistures were estimated, as well as soil density and gross irrigation depths of the treatments [58]. Then, two lysimeters were installed in each experiment with two levels of water retention (35 and 70% of the retention capacity). The mass was measured daily using load cells (model GL 50; Alfa Instrumentos Eletrônicos SA). Data were stored in a datalogger (CR10X Campbell Sci., Logan – USA). The datalogger data were extracted using the PC200W software, maintaining the soil moisture retention levels stable manually every two days for a period of 6 to 18 h.

The water retention level was kept at 70% of the water retention capacity in the two treatments after transplantation. Water deficit began at 30 days with a reduction of the retention capacity to 50% and, after seven days, to 35%. Subsequently, the measurement of masses of lysimeters was performed every two weeks using a digital scale and, whenever necessary, adjusted for possible variations in mass.

The application of Si was carried out via fertigation with an interval of two days using sodium and potassium silicate stabilized with sorbitol at a concentration of 0.0 and 1.8 mmol L^−1^ (113.4 g L^−1^ of Si, 18.9 g L^−1^ of K_2_O, 100 mL L^−1^ of sorbitol, and pH = 11.8). The pH of the solution was adjusted to 6.0 ± 0.5 using a HCl solution (1 mmol L^−1^) and a NaOH solution (1 mmol L^−1^). Potassium balancing was performed in experimental plots without fertigation with Si (0.0 mmol L^−1^), adding KCl in the fertigation solution (8.43 mg L^−1^ of K). To determine the amount of solution with Si in the irrigation, the amount of water lost by evapotranspiration was measured at a time interval of two days. To avoid a greater supply of Si in treatments with no water deficit, the amount of replacement of water loss through evapotranspiration in treatments with water deficit was used as a reference, adjusting this amount to a concentration of 1.8 mmol L^−1^. Therefore, the replacement of water required by evapotranspiration in the water deficit treatments was carried out entirely by the solution with Si, while the treatments in the absence of water deficit were reconstituted by the solution with Si and deionized water.

After 150 days of sugarcane plant sprouting, a cut was carried out 0.1 m from the soil surface of plant shoots, separating it into leaves and stems. The samples were washed under running water, detergent solution (0.1% v/v), HCl solution (0.3% v/v), and deionized water. Then, they were dried in a forced air circulation oven (65 ± 5 ºC) until constant mass. Then, the dry mass of leaves (MSF) and stems (MSC) were determined. Subsequently, the determination of C, N, P, and Si concentrations in MSF and MSC samples was carried out. The determination of C and N were calculated from the dry combustion (1000 ºC) in an elemental analyzer (LECO truspec CHNS) calibrated with the standard LECO 502–278 (C = 45.00%). The determination of P was carried out from the nitric-perchloric digestion and the reading was performed by colorimetry (ammonium metavanadate method) [59]. Finally, Si was determined from alkaline digestion and the reading was conducted by colorimetry with ammonium molybdate [57].

The stoichiometric ratios of leaves and stems were calculated from the quotient of the concentrations of C:N, C:P, N:P, and C:Si and the contents of C, N, P, and Si were estimated from the product of the element concentration and the dry mass. Finally, the use efficiency of C, N, and P were calculated by the ratio of the square of the dry mass and the nutrient content [60].

Data processing was performed using the Python programming language (v. 3.9.7; Python Software Foundation). Tixon outliers test was performed and when outliers were found, the value was eliminated and the new value was estimated by the technique of partial derivatives. Subsequently, normality [61] and homogeneity tests [62] were performed. After preposition tests, an analysis of variance was performed (*p *< 0.05). When significant, results were submitted to Tukey test (*p* < 0.05). Multivariable analyses of hierarchical clusters were conducted and principal components analysis (PCA) was performed. The cluster analysis was based on the similarity coefficient of the Euclidean distance and the connection of the group by the single linkage method. The PCA was determined by the matrix of correlation.

## Data Availability

The datasets generated and/or analyzed during the current study are available from the corresponding author on reasonable request.

## Abbreviations

C: Carbon
CUE: Carbon use efficiency
K_2_O: Potassium oxide
KCl: Potassium chloride
MSC: Dry mass of stems
MSF: dry mass of leaves
N: Nitrogen
NUE: Nitrogen use efficiency
P: Phosphorus
PCA: Principal component analysis
PUE: Phosphor use efficiency
Si: Silicon
